# Population genomics and phylogeography of the boll weevil, *Anthonomus grandis* Boheman (Coleoptera: Curculionidae), in the United States, northern Mexico, and Argentina

**DOI:** 10.1111/eva.13238

**Published:** 2021-05-04

**Authors:** Tyler J. Raszick, C. Michael Dickens, Lindsey C. Perkin, Ashley E. Tessnow, Charles P.‐C. Suh, Raul Ruiz‐Arce, Theodore N. Boratynski, Marcelo R. Falco, J. Spencer Johnston, Gregory A. Sword

**Affiliations:** ^1^ Department of Entomology Texas A&M University College Station TX USA; ^2^ High Performance Research Computing Texas A&M University College Station TX USA; ^3^ Insect Control and Cotton Disease Research Unit USDA‐ARS College Station TX USA; ^4^ Science & Technology USDA‐APHIS Edinburg TX USA; ^5^ USDA‐APHIS Brawley CA USA; ^6^ Cooperating Association of the School of Agricultural Education #13 Gardening Resitencia Argentina

**Keywords:** boll weevil, ddRADseq, phylogenetics, phylogeography, population genetics, population genomics

## Abstract

The boll weevil, *Anthonomus grandis* Boheman (Coleoptera: Curculionidae), is an important pest of commercial cotton across the Americas. In the United States, eradication of this species is complicated by re‐infestations of areas where eradication has been previously successful and by the existence of morphologically similar variants that can confound identification efforts. To date, no study has applied a high‐throughput sequencing approach to better understand the population genetic structure of the boll weevil. Furthermore, only a single study has investigated genetic relationships between populations in North and South America. We used double digest restriction site‐associated DNA sequencing (ddRADseq) to resolve the population genomic structure of the boll weevil in the southern United States, northern Mexico, and Argentina. Additionally, we assembled the first complete mitochondrial genome for this species and generated a preliminary whole genome assembly, both of which were used to improve the identification of informative loci. Downstream analyses revealed two main lineages—one consisting of populations found geographically west of the Sierra Madre Occidental mountain range and the second consisting of populations found to the east—were revealed, and both were sub‐structured. Population geographic structure was consistent with the isolation by distance model, indicating that geogrpahic distance is likely a primary mechanism driving divergence in this species. Boll weevil populations from Argentina were found to be more closely related to the eastern lineage, suggesting a recent colonization of South America by the eastern lineage, but additional sampling across Mexico, Central America and South America is needed to further clarify their origin. Finally, we uncovered an instance of population turnover or replacement, highlighting the temporal instability of population structure.

## 1 INTRODUCTION

Accurately describing the population structure and dynamics of species is fundamental to understanding their geographic distributions and evolutionary history. This information is especially important for integrated pest management, which needs to consider pest evolution (Gassman et al., 2009; Pélissié et al., 2018). For widespread pest species, it is critical to understand broad‐scale patterns of gene flow because inappropriately informed control strategies can be compromised by source‐sink dynamics that could nullify the effects of local suppression (Carrière et al., 2012; Hanski & Gilpin, 1991; Harrison, 1991; Sword et al., 2010; Zaller et al., 2008). Thus, it is important to understand how geography influences gene flow among widespread pest species. In the border regions of the southern United States (US) and northern Mexico, one such geographic barrier to gene flow may be the Sierra Madre Occidental mountain range, which separates Mexico's west coast from the central highlands. The range runs roughly north to south along the west coast of Mexico, from the Arizona‐Sonora border toward where it connects with the Sierra Madre del Sur range in southern Mexico. For management of pest species occurring on both sides of this range, it is critical to understand whether there is gene flow among populations occurring on either side. In this study, we examined the population genetic structure of a cotton pest species that occurs on both sides of this range: the boll weevil, *Anthonomus grandis* Boheman (Coleoptera: Curculionidae). In addition to representative specimens from multiple populations across the species’ North American distribution, we have also sampled populations from South America and inferred the genetic relationship of these populations to their North American counterparts. We also sampled collection localities temporally to assess the stability of the population genetic structure over time.

The boll weevil is a major economic pest of commercially cultivated upland cotton, *Gossypium hirsutum* L. (Malvaceae), across the Americas. Native to Mesoamerica, it is generally accepted that the most recent common ancestor of the boll weevil originated in southern Mexico and Central America and diverged from the sister species, *Anthonomus hunteri* Burke and Cate, during the Pliocene (Alvarado et al., 2017; Burke et al., 1986). The original host plant for the ancestral weevil was probably of the genus *Hampea* Schltdl. (Malvaceae). The weevil underwent at least one host shift to one or more endemic *Gossypium* L. species and later shifted to *G*. *hirsutum* after its cultivation began in the Americas. The species has since expanded its geographic distribution over time, likely in association with the expansion of the cultivation of commercial cotton. In the late 1800s, the boll weevil underwent a northward range expansion through Mexico and eventually across the entire cotton‐producing region of the southern United States (US), where it became an infamous agricultural foe (Burke et al., 1986; Lange et al., 2009). A second range expansion has more recently occurred in South America. First recorded in Venezuela in 1949, the species’ range expanded to Colombia in 1951, Brazil in 1983, Paraguay in 1991, Argentina in 1993, and Bolivia in 1997 (Scataglini et al., 2006). By 2016, the boll weevil had spread as far south as the Argentine province of Santiago del Estero and as far west as the province of Salta. In Mexico, Central America, and South America, the boll weevil remains widely regarded as the most important pest of cotton agriculture.

Boll weevil management in North America is complicated by the existence of morphologically and genetically similar variants that can confound diagnostic efforts (Barr et al., 2013; Burke et al., 1986; Burke, 1968; Fye, 1968; Roehrdanz, 2001; Warner, 1966). This, in turn, inhibits rapid response to weevil outbreaks and limits managers’ ability to identify unmanaged populations that may act as sources for re‐infestations of previously eradicated areas. Further, recent genetic investigations of boll weevil populations have suggested that the current subspecific taxonomy may not accurately describe the reality of the population structure. Classic descriptions of boll weevil variants have generally referred to three forms: (1) the southeastern boll weevil (*A. g*. *grandis*); (2) the Thurberia weevil (*A. g*. *thurberiae*), which has traditionally been regarded as a host‐race associated with Arizona wild cotton, *Gossypium thurberi* Todaro (Malvaceae); and (3) the Mexican boll weevil, an intermediate form that has never been given any formal subspecies designation (Burke et al., 1986; Cross et al., 1975; Warner, 1966). Under this “three‐form” hypothesis, the southeastern boll weevil is described as having a geographic range stretching across the southeastern United States extending south into northern Mexico, east of the Sierra Madre Occidental mountain range, where it overlaps with the Mexican boll weevil variant; the Mexican boll weevil has a U‐shaped distribution throughout much of Mexico's lowlands, with only a slight overlap with the very limited range of the Thurberia weevil in southern Arizona and northern Sonora (Supporting Information A). These subspecific denominations are based primarily on morphological characteristics that are notoriously unreliable and may be labile to diet (Barr et al., 2013; Roehrdanz, 2001). Combined with the overlapping ranges of the three forms, these inadequate morphological descriptions have led to inconsistent application of taxonomic status to boll weevil populations throughout the literature and can cause confusion for management.

Recent research has suggested that the boll weevil variant designated as *A. g*. *thurberiae* may be divergent due to the hypothesized geographic barrier to gene flow of the Sierra Madre Occidental mountain range, rather than due to any host plant association (Alvarado et al., 2017; Kuester et al., 2012). These studies have opposed the three‐form hypothesis altogether, instead proposing a genetic two‐form hypothesis wherein the Thurberia weevil should be regarded as a uniquely host‐associated population of a more widely distributed western genetic lineage whose distribution stretches southward along the western side of the Sierra Madre Occidental mountain range. The second form under this hypothesis consists of populations who are members of an eastern genetic lineage with a distribution east of the Sierra Madre Occidental mountain range and continuing into southern Mexico. The proposed zone of contact for the two forms is along the southern Pacific coast of Mexico, which is part of the historical range from where the species initially expanded its range northward along the eastern and western fronts (Alvarado et al., 2017; Burke et al., 1986; Kuester et al., 2012).

The primary goal of this study was to elucidate the current population genetic structure of *A*. *grandis* across the Americas to better inform management efforts in the United States and northern Mexico. Though a number of studies have investigated the population genetic structure of the boll weevil (Alvarado et al., 2017; Barr et al., 2013; Bartlett, 1981; Kim & Sappington, 2004a, 2004b, 2006; Martins et al., 2007; Roehrdanz, 2001; Scataglini et al., 2000, 2006), none have taken advantage of high‐throughput sequencing technology to generate a multi‐locus dataset that can provide substantially more resolution than classic population genetic markers. Here, we used double digest restriction site‐associated DNA sequencing (ddRADseq, Peterson et al., 2012) to generate a genome‐wide dataset of single nucleotide polymorphism (SNP) markers as a means to better understand spatial and temporal patterns of genetic variation in boll weevil population structure. We generated a preliminary reference genome sequence and used it to inform phylogenetic and population genetic approaches to formally test the two‐form and three‐form hypotheses and resolve the population structure within the resulting lineages. For some populations, we were able to sample in multiple years, allowing for us to not only resolve the spatial structure of populations, but also to evaluate the robustness of that structure over time. Additionally, we evaluated Argentine populations of the species to determine their relationship to the North American lineages. To conclude, we considered the implications of our findings with regard to the current subspecific taxonomy, the international efforts to control the pest populations of the species, and efforts to resolve the population structures of other pest or nonpest species.

## 2 MATERIALS AND METHODS

### 2.1 Specimen sampling

Our sampling regime targeted five main geographic regions. These regions included four commercial cotton production areas: northeastern Argentina (ARG), the lower Rio Grande Valley along the United States–Mexico border (RGV), the Chihuahuan Desert ecoregion in Mexico (CMX), and Sonora, Mexico (SMX). The fifth geographic region was southern Arizona where wild cotton, *G*. *thurberi* is native (AWC). A total of 292 weevil specimens were collected and processed across 20 spatiotemporally distinct collections (Table 1). Weevil specimens from the four commercial production areas were mainly collected using boll weevil pheromone‐baited cone traps (Cross et al., 1969; Cross & Hardee, 1968; Hardee et al., 1971; Tumlinson et al., 1969), whereas those from Arizona were collected directly from *G*. *thurberi* plants using a beat bucket technique wherein branches or whole crowns of plants were shaken into a bucket, dislodging adult weevils into the bottom of the bucket. Insects from all localities were preserved immediately in 95–100% ethanol. Other than during shipping or transportation, all specimens were stored at −80°C until they were removed from storage for DNA isolation. For those collection localities where there were multiple pheromone‐baited cone traps, the midpoint GPS coordinates were determined from the GPS coordinates of the traps using the center of gravity method on the geographic midpoint calculator available at www.geomidpoint.com.

**TABLE 1:** Collection information for all boll weevil specimens in the study

Date	Country	State/Prov.	Locality	Method	Host Plant	Abbreviation	Latitude	Longitude	*N*	*F*_IS_
22‐Sep‐14	Mexico	Sonora	Cajeme	Cone trap	–	SMX‐Caj (2014)	27.4209	−109.9758	5	0.057
22‐Sep‐14	Mexico	Chihuahua	–	Cone trap	–	CMX‐Chi (2014)	28.3431	−105.5720	4	0.1027
23‐Sep‐14	Mexico	Durango	–	Cone trap	–	CMX‐Dur (2014)	26.1229	−103.4147	5	0.2784
12‐Sep‐14	Mexico	Tamaulipas	–	Cone trap	–	RGV‐Tam (2014)	25.8247	−98.0672	16	0.1643
Aug/Sep‐14	USA	Texas	–	Cone trap	–	RGV‐Tex (2014)	26.0713	−97.4655	18	0.1291
28‐Aug‐16	USA	Arizona	Mt. Lemmon	Beat bucket	*G. thurberi*	AWC‐Lem (2016)	32.3262	−110.7004	12	0.066
29‐Aug‐16	USA	Arizona	Sahuarita	Beat bucket	*G. thurberi*	AWC‐Sah (2016)	31.9633	−110.8075	12	0.1227
29‐Aug‐16	USA	Arizona	Highway 83	Beat bucket	*G. thurberi*	AWC‐H83 (2016)	31.9470	−110.6640	12	0.1967
29‐Aug‐16	USA	Arizona	Agua Caliente	Beat bucket	*G. thurberi*	AWC‐Cal (2016)	31.6845	−110.9585	12	0.0594
30‐Aug‐16	USA	Arizona	Bisbee 1	Beat bucket	*G. thurberi*	AWC‐Bi1 (2016)	31.4877	−109.9873	12	0.1534
30‐Aug‐16	USA	Arizona	Bisbee 2	Beat bucket	*G. thurberi*	AWC‐Bi2 (2016)	31.4421	−109.8268	12	0.1341
Jul/Aug‐16	Mexico	Tamaulipas	–	Cone trap	–	RGV‐Tam (2016)	25.8283	−98.0561	34	0.1506
Jul/Aug‐16	USA	Texas	–	Cone trap	–	RGV‐Tex (2016)	26.1594	−97.8234	30	0.0253
Aug/Sep‐17	Mexico	Sonora	Cajeme	Cone trap	–	SMX‐Caj (2017)	27.3086	−109.9939	30	0.0135
7‐Aug‐17	Mexico	Coahuila	–	Cone trap	–	CMX‐Coa (2017)	25.8134	−102.9910	30	0.05
Jun/Jul‐17	Argentina	Chaco	Gral. Pinedo	Cone trap	–	ARG‐Cha (2017)	−27.2533	−61.4942	12	−0.0655
Jun/Jul‐17	Argentina	Chaco	Saenz Peña	Cone trap	–	ARG‐Sae (2017)	−26.8553	−60.4378	8	0.1633
Jun/Jul‐17	Argentina	Salta	–	Cone trap	–	ARG‐Sal (2017)	−25.4256	−63.8483	8	0.0638
Jun/Jul‐17	Argentina	S. del Estero	–	Cone trap	–	ARG‐San (2017)	−29.2397	−62.9083	8	0.1321
Jun/Jul‐17	Argentina	Formosa	–	Cone trap	–	ARG‐For (2017)	−24.6978	−59.4717	12	−0.0817

Note

The “Method” column indicates whether the individuals were sampled from pheromone‐baited cone traps near commercial *G. hirsutum* fields or by beat bucket directly from *G. thurberi*. The “Abbreviation” column denotes the code used for each collection throughout the paper and includes the geographic region of the collection (ARG = Argentina, AWC = Arizona wild cotton, CMX = Chihuahuan Desert ecoregion of Mexico, RGV = Rio Grande Valley, SMX = Sonora, Mexico), the specific collecting locality within the region, and the year of the collection. *N* is the number of individuals analyzed from each collection, and *F*_IS_ is the inbreeding coefficient for each population.

Weevils were first collected in 2014 in Mexico from the cotton‐producing states of Sonora, Chihuahua, Durango, and Tamaulipas, as well as from the lower Rio Grande Valley (RGV) cotton production area in Texas, USA, just north of Tamaulipas along the United States–Mexico border. In 2016, the Texas and Tamaulipas localities were resampled, and an estimated 14–20 generations were expected to have occurred between sampling events. Weevil specimens were also collected in 2016 from six wild cotton (*G*. *thurberi*) localities in southeastern Arizona. In 2017, the Sonora locality was resampled, but for the central Mexico (CMX) geographic region, we collected weevils from Coahuila, Mexico, rather than the previously sampled Durango and Chihuahua localities due to variation in weevil presence from year to year. The Coahuila and Durango localities were only 55 km apart and are both part of a contiguous cotton production region known as La Laguna. These two populations were thus expected to be representative of the same geographic population, though this was not assumed a priori in downstream analyses. Specimens from Argentina were collected in 2017 from five localities in the four cotton‐producing provinces of Chaco, Salta, Santiago del Estero, and Formosa, using cone traps baited with synthetic boll weevil pheromone (Grandlure, Plato Industries Inc.).

### 2.2 DNA isolation, library preparation, and double digest RAD sequencing

The Gentra Puregene Cell and Tissue Kit (Qiagen) was used to isolate genomic DNA from whole weevil specimens. Individuals collected in 2014 were processed and delivered for sequencing in 2015, and individuals collected in 2016 and 2017 were processed and delivered for sequencing in 2017. DNA was isolated from all individuals using the same protocol (Supporting Information B), but with a slight modification from the 2015 batch to 2017. Specifically, specimens sequenced in 2015 were chopped into pieces using sterilized dissecting scissors prior to lysis by proteinase K, whereas those prepared in 2017 were broken into pieces by freezing them in liquid nitrogen and crushing them with disposable pestles. Isolated DNA from all 292 specimens was verified for high molecular weight via electrophoresis on a 1.5% agarose gel. Both methods yielded high quality DNA with fragment sizes greater than 10,000 base pairs.

Genomic DNA isolated from weevils was delivered to the Texas A&M AgriLife Genomics and Bioinformatics Service (TxGen) for purification, library preparation, and sequencing. DNA was purified using the Agencourt AMPure XP purification system (Beckman Coulter) prior to library preparation. Library preparation for the ddRADseq was nearly identical in 2015 and 2017, but 2015 libraries were prepared for a HiSeq 2500 (Illumina), and 2017 libraries were prepared for a NovaSeq (Illumina). To prepare the ddRADseq libraries, purified genomic DNA was digested using the *NlaIII* and *HindIII* restriction enzymes, which were selected by TxGen to optimize the size distribution of DNA fragments such that the number of fragments ranging 250 to 500 base pairs (bp) was maximized. Fragments were size selected using a Pippin Prep (Sage Science) and were then ligated with standard Illumina adapters, multiplexing indexes, and sequencing primers, albeit with a single notable exception; the R1 reads (forward reads; those sequenced in the 5′ direction) were ligated with a custom sequencing primer that contained the 5′ restriction site remnant. In 2015, libraries were sequenced on a HiSeq 2500 using 2x125 sequencing cycles; 2017 libraries were sequenced on a NovaSeq using 2x150 sequencing cycles. Potential differences in sequence batches were addressed during the bioinformatic analyses as described below.

### 2.3 Preliminary reference genome size estimation, sequencing, and assembly

A total of 10 *A. g*. *thurberiae* (7 adults, 2 larvae, and 1 pupae) were collected on December 27, 2019, from the same collection locality as AWC‐Bi2 (2016). Individuals were flash frozen in liquid nitrogen and stored at −80°C. The genome size of each was estimated as described in Johnston et al. (2019). In brief, a single head was placed into 1 ml of Galbraith buffer in a 2 ml Dounce along with the head of a lab strain of *Drosophila virilis* reference standard (1C = 328 Mbp). Nuclei were released by grinding with 15 strokes of the loose “A” pestle at a rate of three strokes every 2 s. The released nuclei were strained through 45 µm nylon mesh, stained with 50 µg/ml propidium iodide for at least 1 h in the cold and dark, then scored for the relative fluorescence of the 2C nuclei from the sample and standard using a CytoFLEX flow cytometer (Beckman/Coulter). DNA content was determined as the ratio of the mean fluorescence of the sample and standard times the 1C amount of DNA in the *D*. *virilis* standard. A minimum of 1000 nuclei were counted for each 2C fluorescent peak, with a CV <2 for each peak.

We generated a preliminary reference genome sequence for the purpose of mapping HTS reads as part of the SNP calling pipeline. To establish an inbred line, founding F_0_ parents were collected in the lower Rio Grande Valley of Texas and reared for three generations at the boll weevil rearing facility at the Texas A&M AgriLife Research and Extension Center in Weslaco, Texas. Six F_3_ progeny were flash frozen in liquid nitrogen and shipped overnight to Dovetail Genomics. The Dovetail team utilized a proprietary hybrid Illumina‐HiRise approach to assemble the genome sequence. Using this approach, Illumina reads generated by whole genome shotgun sequencing are mapped to intermediate‐range scaffolds and long‐range physical maps generated by Chicago (Putnam et al., 2016) and Dovetail's proprietary Hi‐C technology, respectively. Due to limitations in total DNA yield and quality, the Illumina libraries were prepared from an unsexed F_3_ individual, the Chicago library was prepared from an F_3_ male, and the Hi‐C library was prepared from an F_3_ female. Shotgun reads were mapped to the scaffolds using Meraculous version 2.2.4. Run parameters can be found in the Supporting Information C. Genome completeness was assessed using BUSCO version 4.0.2 (Simão et al., 2015) and the insecta_odb10 gene database.

### 2.4 Sequence quality control, SNP calling, and filtering

TxGen provided demultiplexed raw reads and FastQC version 0.11.3 (Andrews, 2010) reports for all 292 specimens. FastQC reports were summarized and reviewed using MultiQC version 1.7 (Ewels et al., 2016). Potential bacterial contamination was filtered out by using Kraken version 1.1 (Wood & Salzberg, 2014) to match sequences to the nonredundant bacterial database hosted by the National Center for Biotechnology Information (NCBI). Trimmomatic version 0.38 (Bolger et al., 2014) was used to ensure that reads from different Illumina runs had a uniform length. A length selected based on the MultiQC report was achieved via removal of the first 10 bp of each sequence and truncating each sequence at 90 total bp.

Mitochondrial genes can become duplicated and inserted into the nuclear genome, forming a nuclear‐mitochondrial DNA sequence (commonly called a “numt” or pseudogene) that may experience different evolutionary pressures than the mitochondrial parent (Bensasson et al., 2001; Grau et al., 2020; Song et al., 2008). If the numt accumulates one or more nucleotide substitutions that are different from the parent, these substitutions can appear as SNP loci in a population genetics dataset. However, these are not true diploid SNP loci because one of the variants is the haploid mitochondrial parent. To remove these false‐positive SNP loci, we assembled a complete mitochondrial genome of *A. g*. *grandis*, and filtered ddRADseq reads that aligned to the mitogenome. The sequence data for this assembly were obtained through whole genome shotgun sequencing of a single individual weevil collected from Tamaulipas in 2014. The weevil selected was the individual among the 2014 collections with the least fragmented DNA, as determined by TxGen using their Pippin Prep. It was not included further as part of this study due to limitation of total DNA yield. Sequences were generated on a HiSeq 2500 using 2x125 sequencing cycles. The open‐source software NOVOPlasty version 2.6.7 (Dierckxsens et al., 2016) was used to separate and extract mitochondrial sequences from the nuclear sequences. Input sequences included 25,781,232 total reads (forward and reverse). A de novo mitochondrial assembly was initiated using the *A. g*. *grandis* cytochrome oxidase subunit 1 gene (GenBank accession number: MF636872.1) as a seed sequence. The mitogenome was annotated using the web‐based program MITOS version 1.0 (Bernt et al., 2013) and visualized with GenomeVx version 1.0 (Conant & Wolfe, 2008). Next, FastQ Screen version 0.12.1 (Wingett & Andrews, 2018) was used to map ddRADseq reads to the mitogenome and to remove them from the dataset.

After quality control, trimming, and filtering were completed, we used the software pipeline dDocent version 2.6.0 to map the remaining ddRADseq reads to the Dovetail Genomics genome assembly and to identify putative SNP loci (Puritz, Hollenbeck et al., 2014; Puritz, Matz et al., 2014). dDocent was run using default parameters with a match score value = 1, mismatch score = 4, and gap opening penalty = 6. VCFtools version 0.1.16 (Danecek et al., 2011) was used to filter the dDocent output in variant call format (VCF) in accordance with the dDocent user guide. Specifically, we removed loci not meeting the criteria of representation in 100% of individuals and a minimum phred quality score of 30, and we removed alleles not meeting the criteria of a minimum minor allele count of three reads per allele, and a minimum minor allele frequency of 0.05. Diploid genotypes with less than 3X coverage were also removed. We then applied filters to remove loci that were out of Hardy–Weinberg equilibrium within populations, nonbiallelic loci, and indels. Finally, we calculated the mean inbreeding coefficient (*F*_IS_) across populations for each locus and removed loci with a mean *F*_IS_ < 0. PGDSpider version 2.1.1.3 (Lischer & Excoffier, 2012) was used to convert the final VCF into the appropriate input formats for phylogenetic analysis (phylip) and population genetics analysis (genepop) when needed, though many analyses simply utilized the VCF directly.

### 2.5 Phylogenetic and population genetic analyses

To create a phylogenetic reconstruction of the sampled populations’ evolutionary history, we accessed the software RAxML version 8.2.10 (Stamatakis, 2014) via the CIPRES Science Gateway version 3.1 (Miller et al., 2010). Geneious 11.0.2 (www.geneious.com) was used to manually check the phylip for abnormalities prior to uploading to CIPRES. Next, PartitionFinder version 2.1.1 (Guindon et al., 2010; Lanfear et al., 2012, 2017), also available on CIPRES, was used to select the best model for reconstructing the tree using a maximized log likelihood criterion. RAxML was run using default parameters with 1000 bootstrap iterations and a GTR+γ model of nucleic acid evolution (the model recommended by PartitionFinder).

RStudio version 1.1.456 (R Core Team, 2018) and some associated packages were used to check the data for potential sources of bias, calculate the *F*‐statistics within and among collections, and to identify genetic populations among collections. R/vcfR version 1.8.0 (Knaus & Grünwald, 2017) was used to load the VCF and prepare objects for use with other packages. R/adegenet version 2.1.1 (Jombart, 2008; Jombart & Ahmed, 2011) was used to create the “genlight” object needed for many of the downstream analyses.

To verify that any observed population genetic structure was not due to sequencing batch effects, we utilized a hierarchical analysis of molecular variance (AMOVA, Excoffier et al., 1992) to test for significant factors contributing to variability in the dataset. Our hierarchical levels were sequencing year and geographic collecting locality. The AMOVA was carried out using R/poppr version 2.8.1 (Kamvar et al., 2014, 2015) with a “farthest neighbor” algorithm and 10,000 permutations. Significance testing was carried out using Monte Carlo resampling with 10,000 permutations with R/ade4 version 1.7‐13 (Bougeard & Dray, 2018; Dray & Dufour, 2007).

It is also possible that some observed differences between populations could be due to differences in relatedness between individuals within each population. To test for this type of potential bias, we calculated pairwise relatedness for every pair of individuals within each population using the software COANCESTRY version 1.0.1.10 (Wang, 2011). COANCESTRY was run using default parameters, and we opted to account for inbreeding. We then used R/car version 3.0‐10 (Fox & Weisberg, 2019) and R/dplyr version 1.0.2 (Wickham et al., 2020) to calculate the mean relatedness and standard deviation within each population and to carry out a Kruskal–Wallis rank test and pairwise Wilcoxon rank sum tests to test for significant differences in the populations. R/ggpubr version 0.4.0 (Kassambara, 2020) was used to visualize the data.

R/genepop version 1.0.5 (Raymond & Rousset, 1995; Rousset, 2008) was used to calculate *F*_IS_ for each population and to estimate gene flow among populations by calculating pairwise *F*_ST_ values (Weir & Cockerham, 1984). Pairwise exact conditional contingency‐table tests for genotypic differentiation (dememorization = 1000, batches = 10, iterations = 500) were also implemented to determine if genetic differences between pairs of collections were statistically significant. To test whether any observed population genetic structure was consistent with an isolation by distance (IBD) model (Rousset, 1997; Wright, 1943), we used option 6 and sub‐option 9 of the web implementation of Genepop version 4.2 (Raymond & Rousset, 1995; Rousset, 2008) to run the Isolde program. Isolde queried the correlation between the semi‐matrix of pairwise *F*_ST_ values and a semi‐matrix of pairwise geographic distances for all pairs of collections in our dataset using a Mantel test with a minimum sample distance of 0.0001 and 10,000 permutations. The values of *F*_ST_ were transformed to *F*_ST_/(1−*F*_ST_). Geographic distances were measured as straight‐line distances (in kilometers) between pairs of GPS coordinates and then transformed by the natural logarithm. The adjusted values of *F*_ST_ and straight‐line distances were extracted from the Isolde output and plotted in Microsoft Excel to calculate the slope and intercept of the linear regression and calculate the *R* ^2^ value.

R/adegenet was used to carry out a principal component analysis and to further group collections into putative genetic populations using a discriminant analysis of principal components. The number of groups was determined de novo using the K‐means clustering algorithm. We tested 1 ≤ K ≤ 21 using all principal components, 100 starting centroids, and 1,000,000,000 iterations per run. The optimal K was selected using the Bayesian information criterion (Supporting Information D). For the discriminant analysis, we used default parameters and retained six principal components and two discriminant functions. R/ggplot2 version 3.2.1 (Wickham, 2016) was used to visualize the spatial clustering of individual genotypes.

The program fastSTRUCTURE version 1.0 (Raj et al., 2014) was used to calculate each sampled individual's probability of assignment to K predetermined genotypic groups where 1 ≤ K ≤ 21. fastSTRUCTURE was run using default parameters. PLINK version 1.07 (Purcell et al., 2007) was used to convert the vcf file into a format that was suitable for input into both programs. The browser‐based program StructureSelector (Li & Liu, 2018) was then used to evaluate the fastSTRUCTURE outputs to choose the optimal K value for our dataset using the “LargeKGreedy” algorithm with 2000 repeats. The optimal K was selected using a maximized marginal likelihood framework (Supporting Information E). CLUMPAK (Kopelman et al., 2015), which is integrated into StructureSelector, was used to visualize individual assignment probabilities.

## 3 RESULTS

### 3.1 Preliminary reference genome size estimation, sequencing, and assembly

The Dovetail Genomics assembly of the boll weevil genome contained 8017 scaffolds spanning 427.92 Mbp. BUSCO results (Table 2) were consistent with a partial genome assembly of 62.86% of our predicted size based on flow cytometry (680.85 Mbp ± 6.68 std. error). The assembly features low fragmentation (scaffold L50/N50 = 8 scaffolds/22.313 Mbp) and high coverage (mean depth 5973X) for the regions of the partial genome that were successfully sequenced. A full report for the final assembly can be found in Supporting Information F.

**TABLE 2:** Number and percentage of insecta_odb10 BUSCOs (Benchmarking Universal Single‐Copy Orthologs) in the preliminary reference genome, indicative of the assembly completeness. Total BUSCO groups are those genes that are expected to be highly conserved across insects

	*N* BUSCOs	% BUSCOs
Complete BUSCOs	852	62.4%
Complete and single‐copy BUSCOs	847	62.0%
Complete and duplicated BUSCOs	5	0.4%
Fragmented BUSCOs	181	13.2%
Missing BUSCOs	334	24.4%
Total BUSCO groups searched	1367	

### 3.2 Sequence quality control, SNP calling, and filtering

TxGen provided a total of 1.42 TB worth of sequence data across the two ddRADseq runs, and the sequences were generally found to be of high quality as evaluated by FastQC and MultiQC. Kraken determined that an average of 2.64% of the sequences per individual were putatively bacterial in origin, and those sequences were removed. Though the second sequencing run yielded sequences that were 150 bp, the first only yielded 125 bp sequences, and so we choose a Trimmomatic truncation length of 90 that was more appropriate for the first run than the second. This resulted in some loss of data but limited allele dropout due to the different sequencing run lengths.

We successfully reconstructed the first complete mitochondrial genome for *A. g*. *grandis* (Supporting Information G). From the initial shotgun sequencing, NOVOPlasty identified 0.35% of the input reads as mitochondrial in origin and assembled 46,138 reads with a 653X average depth of coverage. The assembly consisted of a single, circularized contig with a total sequence length of 17,089 bp and included 22 tRNA genes, 2 rRNA genes, 13 protein‐coding genes, and a major noncoding, AT‐rich control region. These characteristics are typical of coleopteran mitochondrial genomes (Cameron, 2014; Liu, Bian et al., 2016; Ojo et al., 2016; Sheffield et al., 2008), but the overall length is slightly shorter than has been previously reported in boll weevil (roughly 18,000–19,000 bp, Roehrdanz, 2001). The mitogenome assembly was used to filter potential false‐positive SNP loci due to the occurrence of numts in the genome, and we removed at least one ddRADseq locus and a small number of other reads that were present with low coverage.

Our dDocent run identified 116,524 homologous variant loci which were ultimately filtered to 442 SNP loci.

### 3.3 Phylogenetic and population genetic analyses

**FIGURE 1:**
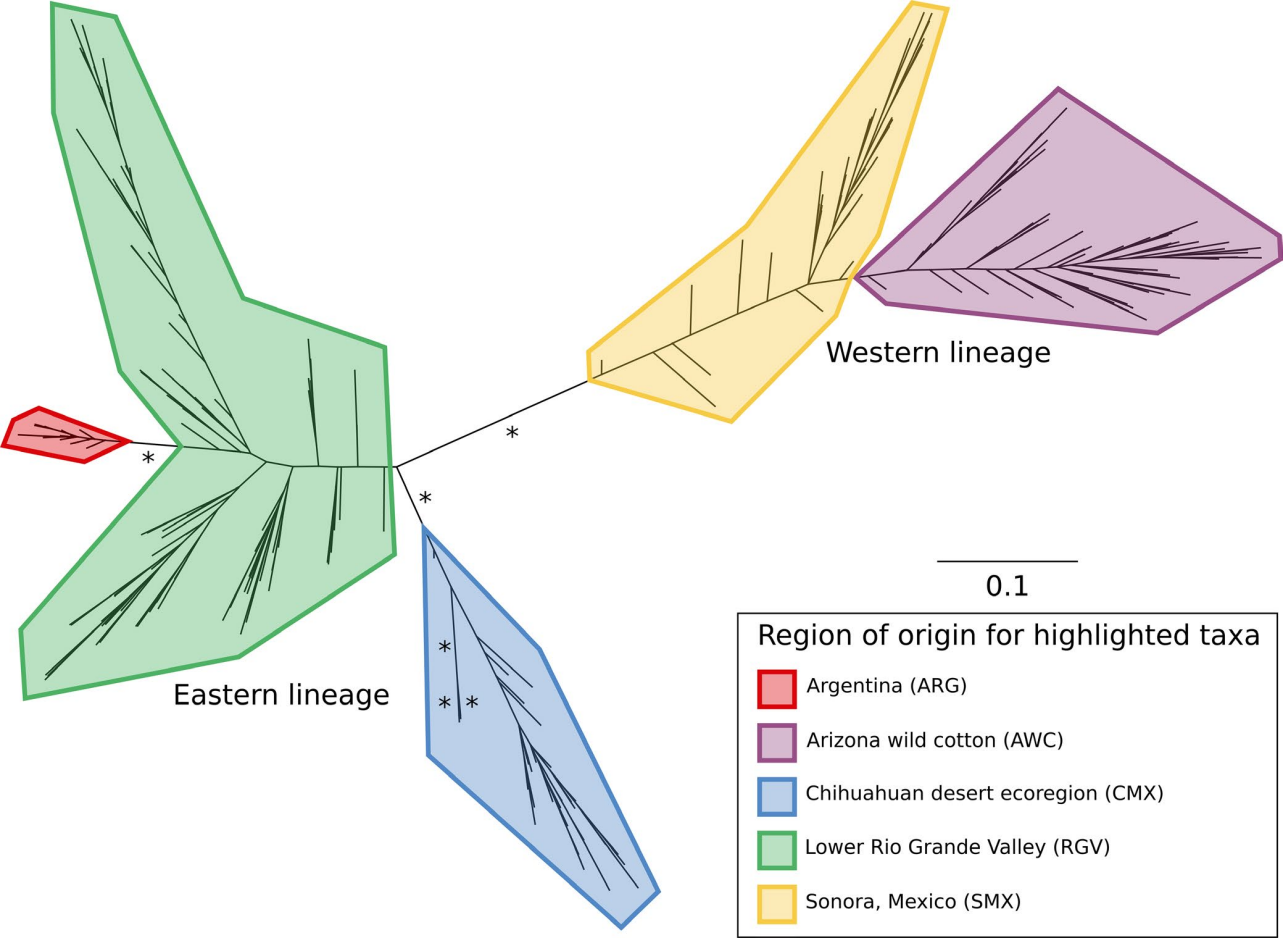
Unrooted RAxML phylogeny of weevils sampled from five geographic regions. Polygonal highlights indicate region origin. Branch lengths represent the number of substitutions per site, and *indicates a branch with ≥95% bootstrap support. The two main divergent lineages that are separated by the long internal branch recapitulate collections of individuals from the western side or eastern side of the Sierra Madre Occidental mountain range

**FIGURE 2:**
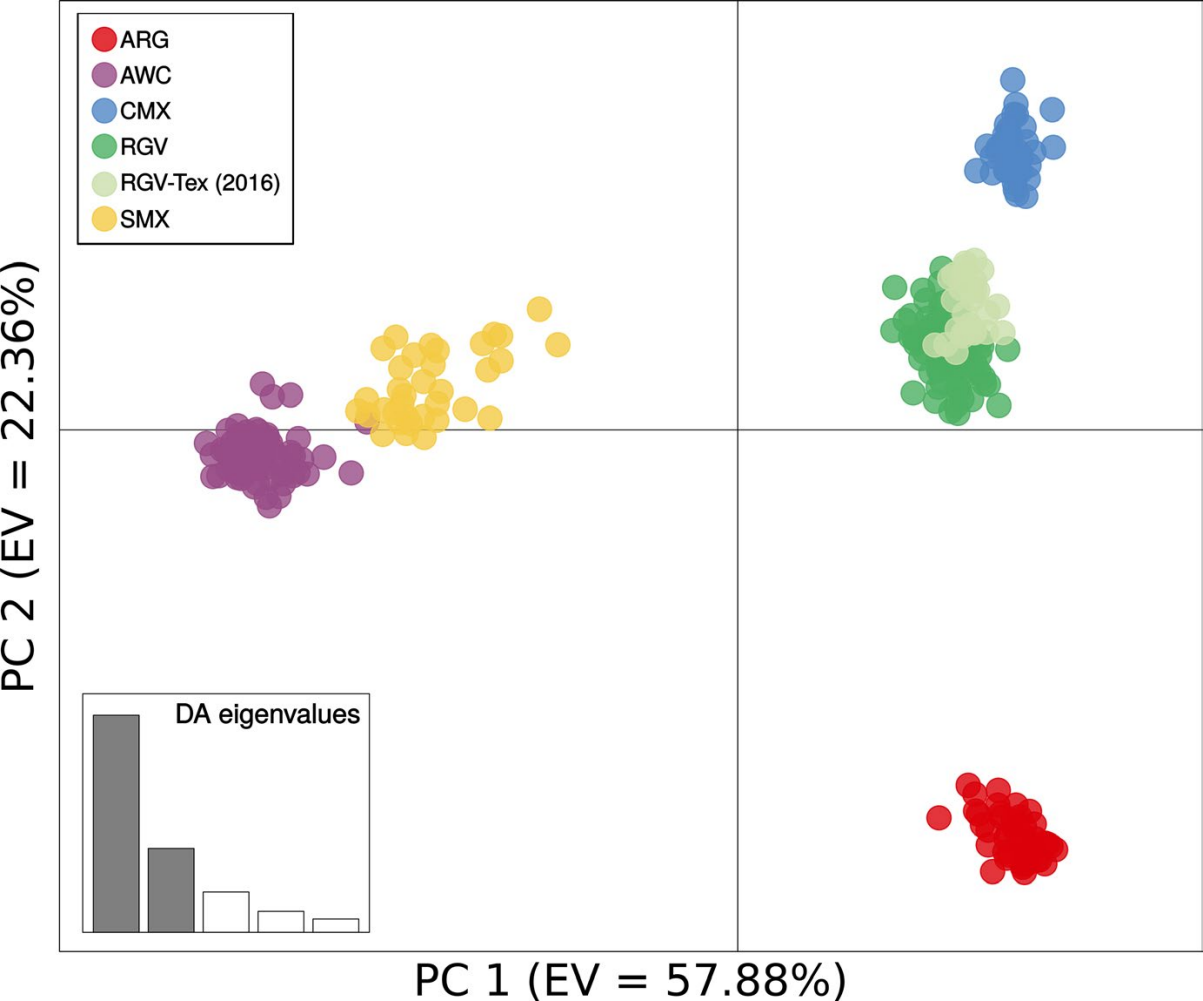
Results of the discriminant analysis of principal components (DAPC). Eigenvalues (EV) of the discriminant analysis indicate the proportion of the observed variation that is explained by the corresponding PC. Individuals are represented by colored dots wherein their color indicates their membership in one of the six identified genotypic groups

**FIGURE 3:**
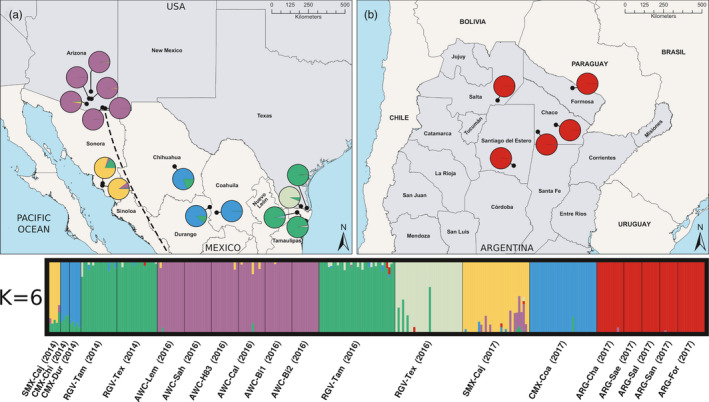
Geographic distribution of sampled populations in North America (panel a) and Argentina (panel b). fastSTRUCTURE results at K = 6 are plotted below the map panels. Each individual's bar illustrates the probabilities of assignment to six genotypic groups. Pie charts overlaid on panels (a) and (b) show population means for probabilities of assignment. Approximate location of the Sierra Madre Oriental mountain range is indicated by the dashed line in panel (a)

**FIGURE 4:**
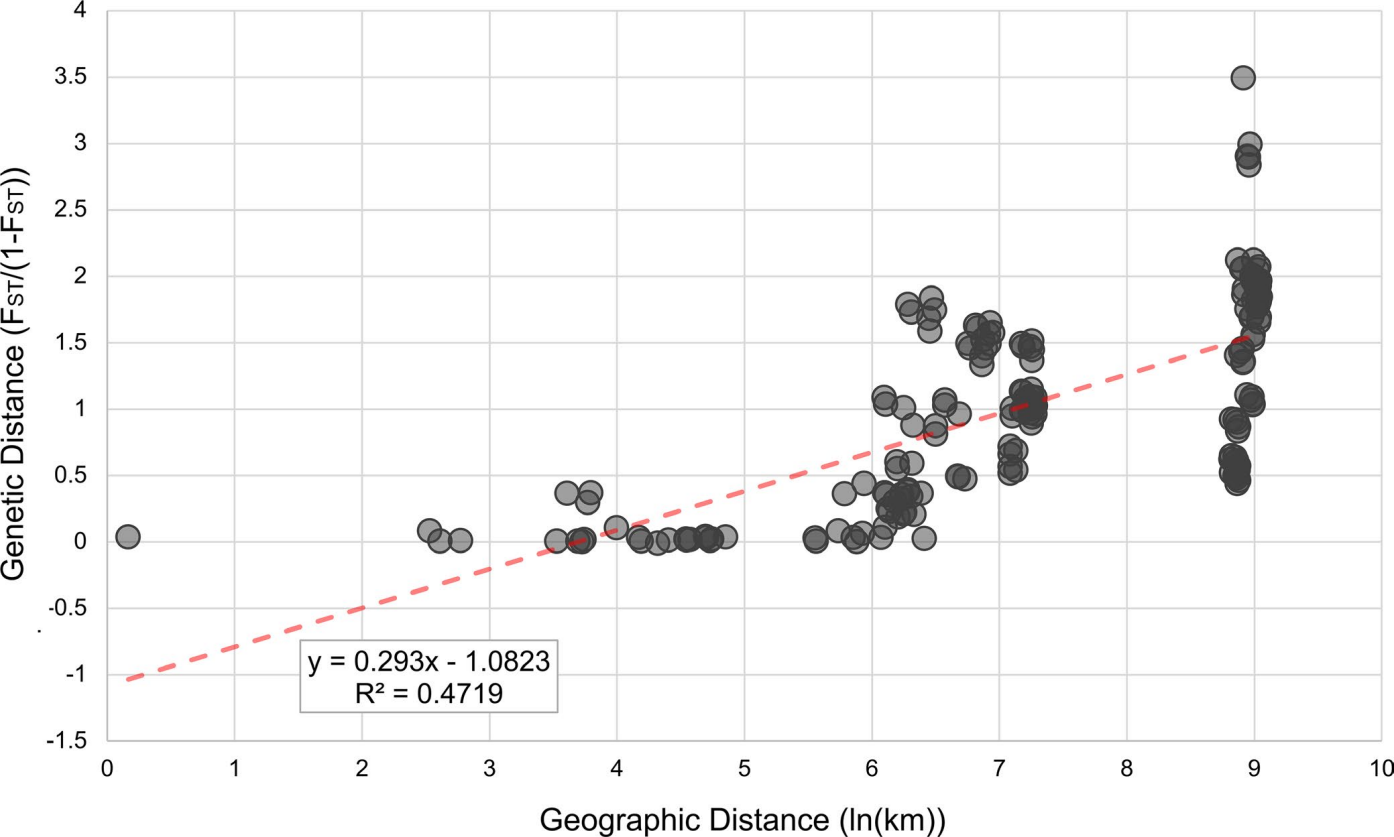
Pairwise genetic distances plotted as a function of pairwise geographic distances. Mantel test for isolation by distance yielded Pr(correlation > observed correlation) = 0.00000 under null hypothesis. Inset shows the equation and *R* ^2^ value for the linear regression

The phylogenetic reconstruction (Figure 1) and the population genetic analyses (Figures 2 and 3) were generally congruent. The results indicated that the sampled collections consisted of two main geographic lineages that were sub‐structured into five (Figure 1) or six (Figures 2 and 3) genetically distinct populations. The population genetic structure was found to be intimately tied to the geographic distribution of the sampled collections, consistent with an IBD model (Figure 4), and there was support for two main geographic lineages distributed on either side of the Sierra Madre Occidental mountain range. In addition to the geographic structure, we also found evidence of temporal instability. Specifically, one collection locality, RGV‐Tex, experienced a population turnover event or replacement wherein nearly all individuals collected in 2016 were genetically distinct from those collected in 2014 (Figures 2 and 3). Our AMOVA analysis indicated that sequencing year was not a significant factor in generating variation between collections (Supporting Information H), so this result was not likely due to any sequencing run bias. Likewise, our relatedness analysis did not indicate any concerning bias in the dataset (Supporting Information I). Instances of high relatedness between individuals were common but not entirely unexpected, given our sampling scheme. Pheromone‐baited cone traps in commercial cotton growing areas are typically used for early detection of new infestations, so collections made from those traps may have been from small founder populations. For AWC collections, weevils typically occurred in isolated patches (often on a single plant), so we may have again sampled small populations wherein some individuals were kin. Though there were indeed pairs of collections that yielded significantly different relatedness as per the Wilcoxon rank sum test (Supporting Information I), there was very high variance in the dataset and no obvious correlation between relatedness and how collections were grouped into genetic populations by the other analyses. For example, the ARG‐Sal (2017) relatedness was found to be significantly different from the ARG‐For (2017) population, but they were consistently grouped together in the subsequent population genetic analyses. Conversely, none of the collections sampled in 2014 were found to have significantly different relatedness, but there were still found to be three distinct genetic populations that year.

Pairwise calculations of *F*_ST_ indicated higher levels of divergence between collections originating from different geographic regions and lower levels of divergence between collections originating from the same region (Supporting Information J, K). The Mantel test for IBD indicated that *F*_ST_ was indeed significantly correlated with geographic distance, and the associated *R* ^2^ value indicated that 47.19% of the variation in the dataset could be explained by IBD (Figure 4).

Our principal components analysis showed that individuals originating from any one collection clustered with other individuals from the same collection, and collections originating from any one geographic region clustered with other collections from the same geographic region (Supporting Information L). Collections clustered on either end of the PC 1 axis divided into two geographic groups: one west of the Sierra Madre Occidental mountain range and one to the east that also included the Argentine collections. When we applied the discriminant analysis to the principal components, we recapitulated that arrangement of individuals in space along the first and second axes (Figure 2). The K‐means clustering analysis identified six statistically distinct genetic clusters (Supporting Information D). These six clusters almost exactly recapitulated the five geographic regions that were originally targeted during sampling; the lone standout was the RGV‐Tex (2016) collection which was identified as a sixth genetically distinct group, different from other collections in the RGV sampling region (Figure 2).

The population assignment probability test was consistent with the result of the discriminant analysis of principal components. As in the discriminant analysis, the optimal K = 6 (Supporting Information E), individuals from the same collection were predominantly assigned to the same genetic group, and collections grouped mainly according to the geographic region from where the collection was sampled (Figure 3). The exception was, again, the RGV‐Tex (2016) collection wherein individuals from that collection were assigned to a unique genotypic group, distinct from all other collections, including others from the RGV geographic region. However, there were four individuals that showed roughly 50% or less probability of assignment to the other RGV group (Figure 3). ARG collections displayed little to no evidence of shared gene flow with any other region. In 2014, there was evidence of introgression of the RGV genotypic group into the CMX and SMX regions, but that signature was not observed in later years. Instead, the SMX region showed some shared probability of assignment with the AWC collections.

## 4 DISCUSSION

We have generated the first partial whole genome sequence and the first complete mitochondrial genome assembly for *A*. *grandis*. Our whole genome assembly, which captures roughly two‐thirds of the estimated genome size, remains a work in progress and should be considered as such. Nonetheless, the portions of the genome that we have assembled are of high quality and are reliable. Using this assembly as a reference for our SNP calling likely had the effect of reducing the total number of loci detected due to ddRADseq reads not matching any part of the reference. Nonetheless, the reads and reference qualities are high, and we used very stringent filters, so the loci we did obtain for subsequent analyses are not compromised due to the partial assembly. Our mitogenome assembly was also somewhat shorter than expected. Nonetheless, the assembly has high completeness, contiguousness, and coverage depth. So, this discrepancy in length is likely attributable to the assembly software underestimating the number of repeats in the AT‐rich control region. The position of the tRNA‐isoleucine (trnI) has undergone a rearrangement into the middle of the control region, differing from the ancestral arrangement found in most insects. Similar rearrangements have been found in *Sitophilus oryzae* and *S*. *zeamais* (Ojo et al., 2016), and complete losses of the trnI have been documented in other weevils (Liu, Gao et al., 2016; Nan et al., 2016; Song et al., 2010; Tang et al., 2017). Further investigation of these rearrangements and losses in the Curculionoidea may be warranted, as they may be taxonomically informative for higher level phylogenetics.

Overall, the results of our phylogenetics and populations genetics analyses indicated that the sampled individuals represented two main boll weevil lineages and that those lineages were highly sub‐structured. The genetic structure was intimately tied to the geography of the populations. There was strong support for IBD as a mechanism for divergence. We also found evidence of temporal instability, suggesting that the population structure could be labile to time.

### 4.1 Revisiting the two‐form and three‐form hypotheses with implications for taxonomy

Two opposing hypotheses of boll weevil variation have been described in the literature: (1) a three‐form morphological hypothesis wherein there exists a southeastern boll weevil (*A. g*. *grandis*), a Thurberia weevil (*A. g*. *thurberiae*), and an intermediate Mexican boll weevil; and (2) a two‐form genetic hypothesis wherein there exists a distinct lineage distributed west of the Sierra Madre Occidental mountain range and a distinct lineage distributed to the east and stretching down into Central and South America. The results of our study were more consistent with the two‐form hypothesis, though it does not fully describe the genetic variation we observed across all sampled populations. There are two primary pieces of evidence that supported this conclusion: (1) the long internal branch separating the two main lineages in the unrooted phylogenetic tree (Figure 1) and (2) the spatial arrangements of the collections along the axis of the first discriminant function (Figure 2).

Though both the phylogenetic reconstruction and the discriminant analysis of principal components pointed to further substructure in both lineages, there is clear evidence for a major genetic divide between the western and eastern lineages. In the phylogenetic reconstruction (Figure 1), the internal branch separating the western and eastern lineages has strong bootstrap support and is the longest span between internal nodes. Though there are other branches with similar support, particularly in the eastern lineage, these branches are not nearly as long. A congruent pattern was observed in the discriminant analysis of principal components. In that analysis, the discriminant function of principal component 1 explained 57.88% of the observed variation. Collections from the western and eastern sides of the Sierra Madre Occidental mountain range are positioned in the coordinate plane space on either end of the principal component 1 axis (Figure 2), suggesting that this axis captures the effect of this geographic barrier to gene flow. Pairwise *F*_ST_ values also generally supported this conclusion, as values tended to be lower when comparing populations from within the same lineage and higher when comparing collections from different lineages (Supporting Information J, K). However, it should be noted that the *F*_ST_ values were generally very high and may be inflated due to the Weir‐Cockerham sensitivity to disparate sample sizes (Bhatia et al., 2013). Very large *F*_ST_ values (>0.5) are typically indicative of species‐level differentiation or greater, and despite some consistency with previous measurements made for boll weevil populations (Alvarado et al., 2017; Kim & Sappington, 2006; Scataglini et al., 2000), such levels are not typically expected within a single species. Nonetheless, our *F*_ST_ values are relatively consistent with our other analyses, and other types of genetic markers have yielded similarly high estimates (Alvarado et al., 2017; Kim & Sappington, 2006; Scataglini et al., 2000), so it remains possible that boll weevil populations are highly divergent in a way that warrants further exploration. Regardless, there is little support for the Thurberia weevil, *A. g*. *thurberiae*, to warrant subspecific taxonomic status. Though the authors recognize the unique association of some populations in the western lineage with *G*. *thurberi*, those populations, in our study, were not more distinct from populations infesting commercial cotton in Sonora than populations in the lower Rio Grande Valley were from those in the Chihuahuan Desert. If those *G*. *thurberi* populations warrant subspecific status, then so should many of the other sampled populations. Despite the host association in those populations, their genetic divergence from other populations could just as easily be explained by IBD (Figure 4). It is also critical to consider that other populations of boll weevils that we did not sample may also utilize alternative host plants. In addition to other species of *Gossypium*, boll weevil has been documented in association with other Malvaceae including *Hampea* spp., *Cienfuegosia* spp., and *Thespia populnea* (Burke et al., 1986; Cross, 1973; Cross et al., 1975). Because our samples were mostly obtained from areas of commercial cotton production, we may have neglected populations associated with these alternative hosts that may act as reservoirs of additional genetic diversity. Considering the U‐shaped distribution of the species in North America, we have not sampled a potentially important region: the southern Pacific coast of Mexico. At the northern ends of the range, the western and eastern lineages are likely evolving in near complete allopatry due to the geographic barrier to gene flow created by the Sierra Madre Occidental mountain range. However, in southern Mexico, where there may be a zone of contact for the eastern and western lineages, there may be intermediate genetic populations. Any proper reassessment of the subspecific taxonomy should consider this geographic gap in our sampling as well as the diversity of alternative hosts that occur throughout the southern vertex of the species’ parabolic North American range.

### 4.2 On the origin of the South American range expansion

Consistent with Scataglini et al. (2006), we found that Argentine boll weevils were more closely related to, and likely derived from, the eastern boll weevil lineage in North America as opposed to the western. Low levels of genetic differentiation among the Argentine populations suggested a single, contiguous population. Consistent with IBD, the vast geographic distances between ARG collections and the other sample regions were correlated with large genetic distances (Supporting Information J, K), and there was very little evidence of gene flow when compared to those other collections (Figures 2 and 3). Though we could confidently infer that Argentine populations were likely founded by migrants from the eastern lineage, it was not possible to discern the absolute source population. It was also not possible, given the current geographic sampling, to ascertain whether intermediate genotypes existed between the eastern boll weevil in North America and the Argentine boll weevil. Nonetheless, it is considered likely that such populations have arisen in other parts of South America that produce commercial cotton during the range expansion. More extensive geographic sampling of boll weevil populations across both southern Mexico and Central and South America will be critical to fully understanding the origin and path of the boll weevil's extensive range expansion.

### 4.3 Considerations for boll weevil management

Our results, based largely on weevils from commercial cotton, showed that there was a large amount of genetic diversity across geographic sampling regions. However, within geographic sampling regions, diversity was much lower, suggesting that in a given region, there is typically a single, contiguous population (Figures 2 and 3). Management and ongoing eradication efforts must recognize that when an area is dominated by such a population, control efforts must be implemented at the area‐wide scale, as local management efforts will be ineffective if the population can re‐establish from nearby source populations with the same genetic profile. This is especially important when considering the role of alternative host plants as a reservoir host for boll weevil. In the western lineage, weevils infesting *G*. *thurberi* in Arizona were not so different from those infesting commercial cotton in Sonora (Figures 1 and 2), and there was evidence of introgression of the AWC genetic group into the SMX collection in 2017. This suggests that, at least, these populations are geographically close enough to interbreed under the right conditions, and that, at worst, could directly provide migrants with the ability to infest commercial cotton. While management cannot realistically control populations in natural areas, this insight should be considered when developing trapping schemes for monitoring. In the lower Rio Grande Valley, managers must acknowledge the contiguity of boll weevil populations along the United States–Mexico border and that effective management will require a coordinated international effort to successfully combat this pest.

More broadly and perhaps most critically, managers must recognize the reality of the temporal instability of pest population genetic structure (Choi et al., 2011; Dinsdale et al., 2012; Lainhart et al., 2015; Raszick et al., 2020). In this study, we discovered a case of population replacement or turnover in the RGV‐Tex sampling locality from 2014 to 2016 (Figures 2 and 3). Though natural processes could certainly contribute to such temporal instability, it would be unwise to neglect the potential influence of management. In any area where boll weevil populations are infesting commercial cotton, those populations are likely to be subjected to a strong selection pressure due to active management or eradication efforts. For example, along the United States–Mexico border where infestations are frequent, cotton is treated with malathion, an organophosphate insecticide, on a regular basis. This is itself a strong selection pressure that likely contributes to genetic bottlenecks or potentially local extinctions, which could allow for recolonization events and founder effects. In the latter case, management at the area‐wide scale would require a coordinated, international effort to prevent recolonization by other nearby infestations with the same genetic profile. Again, wild host reservoirs and volunteer cotton could act as sources for re‐infestation, so it is essential that managers make every effort to remove volunteer cotton and consider wild host reservoirs in their monitoring schemes. Future population genetic research in this species should take care to explicitly sample these alternative hosts.

Genetic bottlenecks could be particularly threatening to effective management if the surviving individuals do so due to resistance to management. Were this the case, it could lead to a selective sweep. Fortunately, in the lower Rio Grande Valley, boll weevil populations have remained susceptible to malathion, so a selective sweep due to management is not expected to have caused the population turnover we have documented here. Nonetheless, a turnover event or replacement does appear to have occurred, which highlights the dynamic nature of the population genetic structure even within a single growing region. Managers in any growing region would be best served by a robust understanding of where genetic populations occur and what reservoirs exist that could possibly contribute to re‐infestations. Population genetic approaches have previously been effective for determining the sources of re‐infestations (Barr et al., 2013; Kim et al., 2006, 2008). It is imperative that future studies consider the possibility that population structure can be influenced by yearly changes in habitat quality or by management and eradication programs themselves, and studies may need to be repeated to monitor for potential shifts in the population structure.

### 4.4 Implications for population genetic studies and management of other pest species

Temporal instability of population genetic structure is not a novel discovery. Studies of economically or ecologically important fish species have long monitored changes in allele frequencies over time (Garant et al., 2000; Glover et al., 2012; Karlsson & Mork, 2005; Skaala et al., 2006), and the phenomenon has not been ignored in entomological research. Local genetic turnovers similar to the one observed in this study have been previously documented on comparable time scales in boll weevil populations in parts of Texas and Mexico using microsatellites (Choi et al., 2011), as well as in other insect pest species (Dinsdale et al., 2012; Lainhart et al., 2015; Raszick et al., 2020). Temporal instability, regardless of whether it is due to a natural process or due to management, must be considered for long‐term effectiveness of area‐wide management. As populations change, so too may the patterns of migration and divergence. This may be critical for addressing source‐sink dynamics in cropping systems or when monitoring for resistant populations. Even for nonpest species, regular monitoring of the population genetic structure over time could provide insights for conservation or for the effects of habitat loss or climate change. At the very least, population genetics studies should recognize that population structure is dynamic, so sampling only a single point in time is akin to taking a snapshot. When possible, populations should be resampled over multiple time points, and the design of the study should allow for additional data to be incorporated as future populations are resampled, enabling comparisons of structure over time.

## CONFLICT OF INTEREST

The authors have no conflict of interest to declare.

## AUTHOR CONTRIBUTIONS

TJR carried out the majority of the analyses and wrote the manuscript. CMD provided custom scripts and generally supported the bioinformatics used for the population genetic analyses. LCP conducted the mitochondrial genome assembly and annotation and the relatedness analysis. AET contributed to the bioinformatics and downstream analyses in R. CPCS, RRA, TNB, and MRF coordinated and supported sample acquisition and contributed intellectually. JSS supported the genome size estimation. GAS provided resources and laboratory space for the study, collected samples, helped write the manuscript, and contributed intellectually. All authors were given opportunity to review and contribute to the manuscript before submission into consideration for publication.

## Supporting information

Supplementary Material

## Data Availability

The full mitochondrial genome sequence for *A. g*. *grandis* used in this study has been made available through the NCBI GenBank under accession number MT319132, and raw reads generated by ddRADseq have been made available through the NCBI Sequence Read Archive (SRA) under BioProject ID PRJNA623635. The preliminary whole genome assembly that was ultimately used for ddRADseq read mapping can be made available upon request to the authors. Unix and R scripts used for the quality control and analysis of ddRADseq data can be found on GitHub: https://github.com/tjraszick/boll_weevil_pop_gen.
