# Supplementary material for: Population genomics and phylogeography of the boll weevil, Anthonomus grandis Boheman (Coleoptera: Curculionidae), in the United States, northern Mexico, and Argentina
Source: Evol Appl. 2021 May 4;14(7):1778–93. doi: 10.1111/eva.13238 (PMC8288010; doi:10.1111/eva.13238)
Supplement: Supplementary file 1 — Supplementary Material [file EVA-14-1778-s001.docx]

**Supplemental Information for:**

**Population genomics and phylogeography of the boll weevil, *Anthonomus grandis* Boheman (Coleoptera: Curculionidae), in the United States, northern Mexico, and Argentina**

Tyler J. Raszick, C. Michael Dickens, Lindsey C. Perkin, Ashley E. Tessnow, Charles P.-C. Suh, Raul Ruiz-Arce, Theodore N. Boratynski, Marcelo R. Falco, J. Spencer Johnston, and Gregory A. Sword

**Table of Contents:**

| **Supplementary Material A:** Distribution map of the three morphological boll weevil forms | Page 2 |
| --- | --- |
| **Supplementary Material B:** DNA purification protocol | Page 3 |
| **Supplementary Material C:** Meraculous run parameters for the Dovetail Genomics HiRise Assembly | Page 5 |
| **Supplementary Material D:** Bayesian Information Criterion  scores for K-means clustering analysis where 1 ≤ K ≤ 21. | Page 6 |
| **Supplementary Material E:** Marginal likelihood plot for  fastSTRUCTURE runs where 1 ≤ K ≤ 21 | Page 7 |
| **Supplementary Material F:** Dovetail Genomics  assembly report for reference genome assembly | Page 8 |
| **Supplementary Material G:** Circularized  mitochondrial genome of *A. g. grandis* | Page 12 |
| **Supplementary Material H:** Analysis of Molecular Variance  (AMOVA) | Page 13 |
| **Supplementary Material I:** Box and whisker plot of  relatedness within populations | Page 14 |
| **Supplementary Material J:** Semi-matrices of  pairwise linear distance and genetic distance | Page 15 |
| **Supplementary Material K:** Summarized pairwise F_ST_ table | Page 16 |
| **Supplementary Material L:** Results of principal  components analysis as performed using R/adegenet | Page 17 |

**
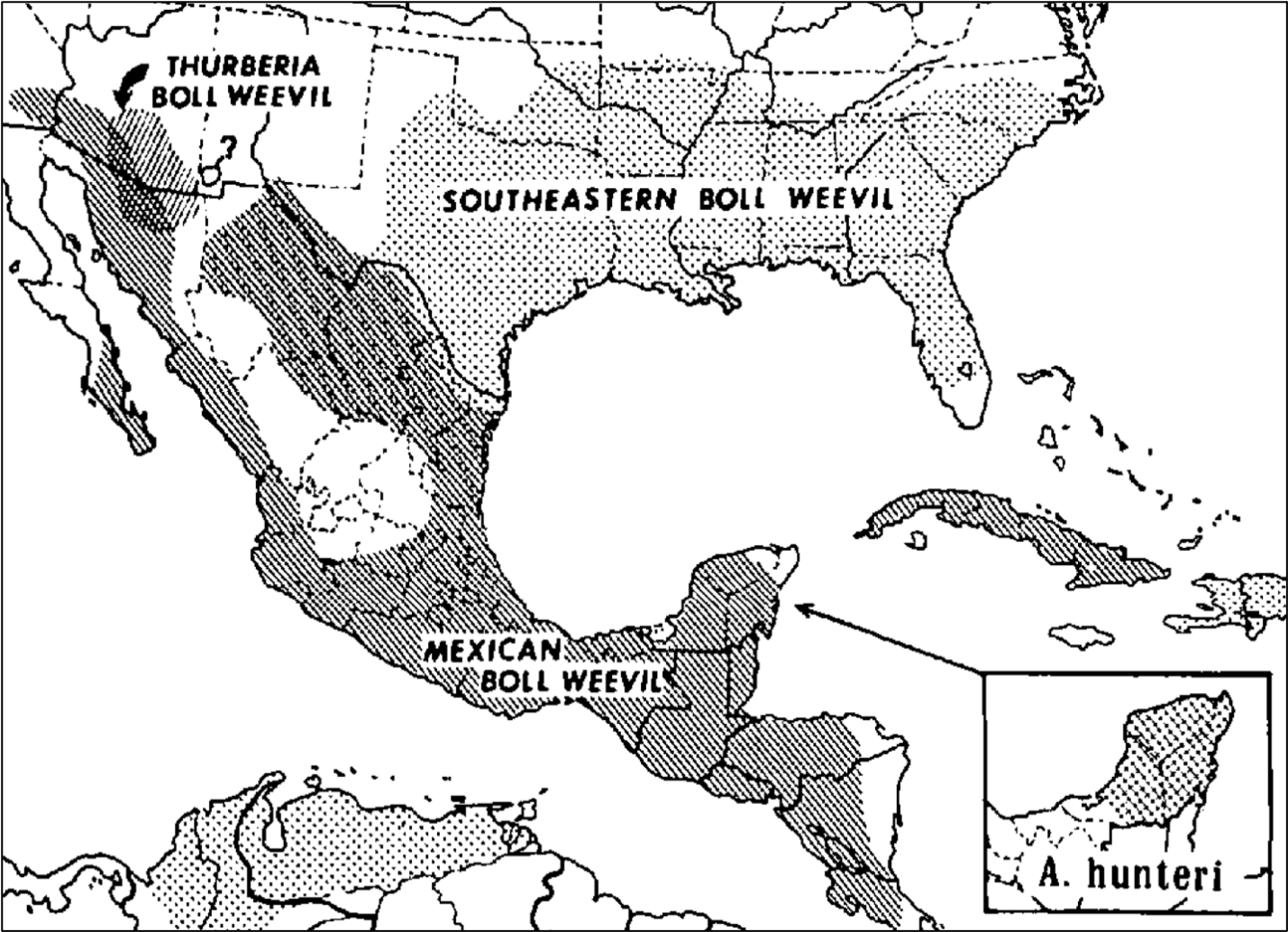
**

**Supplementary Material A:** Map adapted from Burke et al. (1986) showing the distributions of the three morphological forms of boll weevil described under the three-form hypothesis.

**Supplementary Material B:** DNA purification from ethanol-preserved boll weevil using the Gentra Puregene Tissue Kit

Equipment and Reagents not provided with Puregene Kit

1. 1.5 ml microcentrifuge tubes
2. 3, 200 mL beakers with 2% bleach, ethanol, and pure water
3. Sterilized plastic pestles
4. Incubation block
5. Foam cooler
6. Liquid nitrogen
7. Pipets and pipet tips
8. Cold 100% isopropanol
9. Cold 70% ethanol
10. Microcentrifuge
11. Crushed ice
12. Glycogen Solution

Sample Preparation – Day 1

1. Preheat an incubation block to 55°C.
2. Sterilize plastic pestles and soft forceps using 1 minute soaks in 2% bleach, ethanol, and pure water.
3. Briefly blot excess ethanol from specimens on clean absorbent paper.
4. Add 1 whole BW each to a labeled 1.5 ml microcentrifuge tube.
5. Freeze the micro centrifuge tubes in liquid nitrogen until bubbling subsides.
6. Homogenize each tissue using a sterilized plastic pestle.
   1. Add 600 ul of Cell Lysis Solution to each tube and remove the pestle, taking care to leave all tissue in the tube.
7. Add 1.5 ul of Puregene Proteinase K to each tube.
8. Mix by inverting 25 times.
9. Centrifuge for 5 seconds at 16,000 x g.
10. Incubate at 55°C for overnight or until tissue has completely lysed.
11. Invert tube periodically during incubation.
    1. If tissue is not completely lysed after overnight digestion, add 1.5 ul of Proteinase K and incubate for another 3 hours.

DNA Extraction – Day 2

1. Preheat incubation block to 37°C. It is better to use a second heating block if possible, as a cold block will heat to 37°C much more quickly than a block at 55°C will take to cool down.
2. Add 3.0 ul of RNase A solution to the sample.
3. Mix by inverting 25 times.
4. Incubate for 30 minutes at 37°C.
   1. During this incubation, grind a full tray of ice and put it into the foam cooler for upcoming sample incubation.
5. Incubate on ice for 1 minute to quickly cool the sample.
6. Add 200 ul of Protein Precipitation Solution
7. Mix thoroughly by shaking vigorously for 20 seconds.
8. Centrifuge for 3 minutes at 16,000 x g.
9. Incubate for 5 minutes on ice.
   1. During this incubation, pipet 100 ul of isopropanol into a clean 1.5 ml microcentrifuge tube.
10. Centrifuge for 3 minutes at 16,000 x g.
11. You will see a protein pellet that has formed at the bottom of the sample. Without disturbing this pellet, carefully pour the supernatant from the sample into the fresh tube with the 600 ul of isopropanol.
12. Add 1.0 ul of Glycogen Solution to the sample.
13. Mix by inverting gently 50 times.
14. Centrifuge for 5 minutes at 16,000 x g.
15. The DNA may now be visible as a small white or nearly clear pellet at the bottom of the tube. Carefully pour off the supernatant, and drain the tube completely by inverting on a clean piece of absorbent paper, taking care that the pellet remains in the tube.
16. Let tube air dry for 5 minutes.
17. Add 600 ul of cold 70% ethanol to the sample.
18. Invert several times gently to wash the DNA pellet.
19. Centrifuge for 3 minutes at 16,000 x g.
20. Again, carefully pour off the supernatant, and drain the tube completely by inverting on a clean piece of absorbent paper, taking care that the pellet remains in the tube.
21. Allow tube to air dry for 20 minutes or dry using a vacu-fuge (10 minutes at 30°C).
    1. During this step, preheat an incubation block to 65°C.
22. Add 50 ul of DNA Hydration Solution.
23. Shake the sample vigorously for 5 seconds to mix.
24. Centrifuge for 15 seconds at 16,000 x g.
25. Incubate at 65°C for 1 hour.
26. Place on orbital shaker and shake gently overnight at room temperature. Ensure cap is tightly closed to avoid leakage.

Quality Control – Day 3

1. Use a spectrophotometer to obtain concentration and purity estimates.
2. Run sample on an electrophoresis gel to determine relative integrity of DNA.

**Supplementary Material C:** Meraculous run parameters for the Dovetail Genomics HiRise Assembly

mer_size: 109

local_max_retries: 0

num_prefix_blocks: 10

min_depth_cutoff: 9

use_cluster: 0

local_num_procs: 31

local_max_memory: 226

diploid_mode: 1

no_strict_haplotypes: 1

lib_seq: /docker/dovetailgenomics/projects/SWO435_Anthonomus_grandis/denovo/trimmed/DTG_SG_171_hiseq_run1/*_R1.pt*,/docker/dovetailgenomics/projects/SWO435_Anthonomus_grandis/denovo/trimmed/DTG_SG_171_hiseq_run1/*_R2.pt* SG171 418 99 146 0 0 1 1 1 0 0

lib_seq: /docker/dovetailgenomics/projects/SWO435_Anthonomus_grandis/denovo/trimmed/DTG_SG_170_hiseq_run1/*_R1.pt*,/docker/dovetailgenomics/projects/SWO435_Anthonomus_grandis/denovo/trimmed/DTG_SG_170_hiseq_run1/*_R2.pt* SG170 510 99 146 0 0 1 2 1 0 0

genome_size: 0.6252

gap_close_aggressive: 1

**Supplementary Material D:** Bayesian Information Criterion scores for K-means clustering analysis where 1 ≤ K ≤ 21.

**Supplementary Material E:** Marginal likelihood plot for fastSTRUCTURE runs where 1 ≤ K ≤ 21.

**Supplementary Material F:** Dovetail Genomics report for final Anthonomus grandis genome assembly.

Estimated physical coverage (10-10,000 kb pairs): 5,973.17X

|  | Input Assembly | Dovetail HiRise Assembly |
| --- | --- | --- |
| Total Length | 427.45 Mb | 427.92 Mb |
| L50/N50 | 230 scaffolds; 0.363 Mb | 8 scaffolds; 22.313 Mb |
| L90/N90 | 1,725 scaffolds; 0.039 Mb | 17 scaffolds; 13.921 Mb |


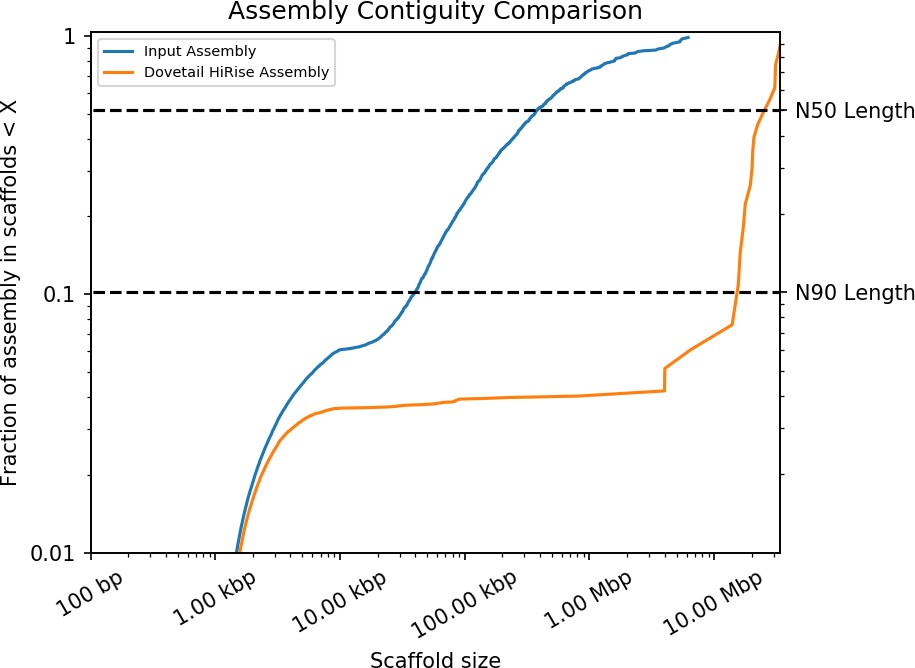


A comparison of the contiguity of the input assembly and the final HiRise scaffolds. Each curve shows the fraction of the total length of the assembly present in scaffolds of a given length or smaller. The fraction of the assembly is indicated on the Y-axis and the scaffold length in basepairs is given on the X-axis. The two dashed lines mark the N50 and N90 lengths of each assembly. Scaffolds less than 1 kb are excluded.


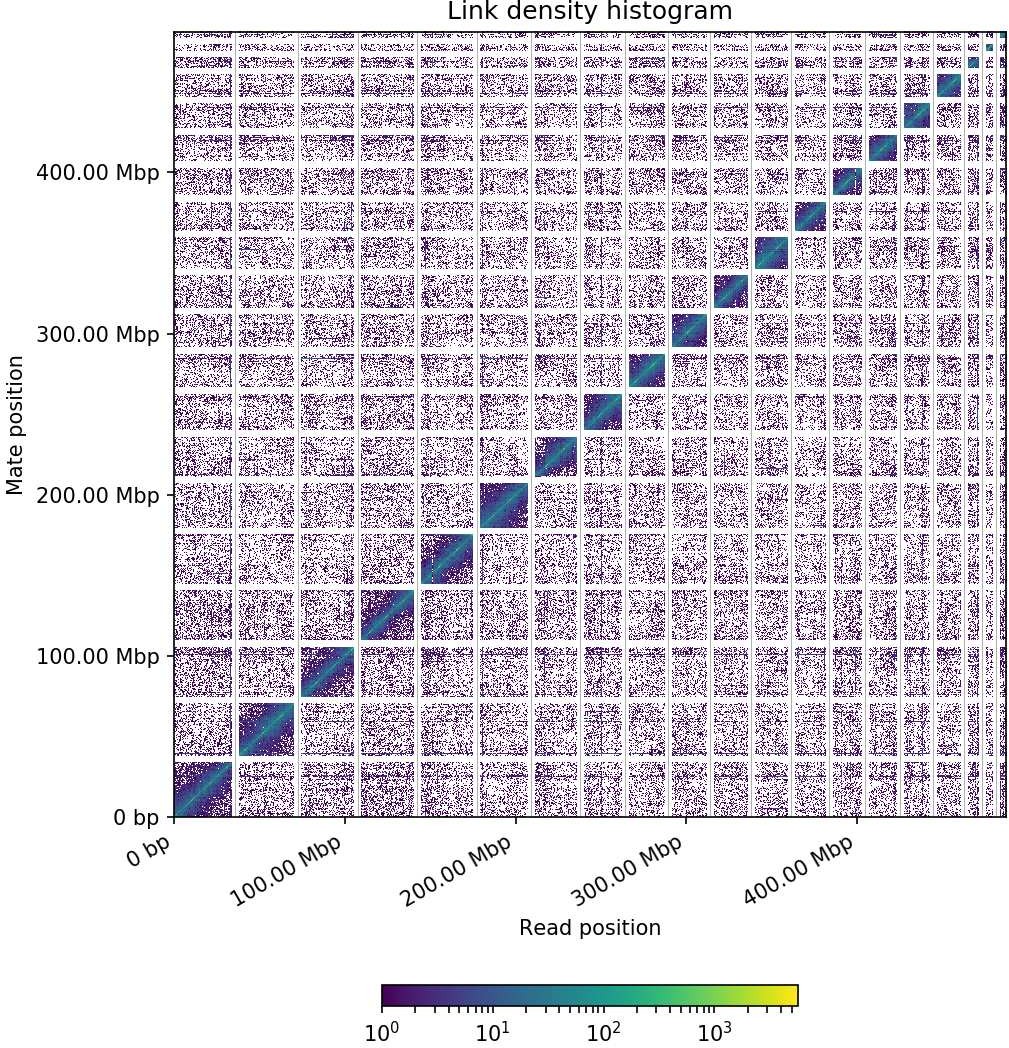


In this figure, the x and y axes give the mapping positions of the first and second read in the read pair respectively, grouped into bins. The color of each square gives the number of read pairs within that bin. White vertical and black horizontal lines have been added to show the borders between scaffolds. Scaffolds less than 1 Mb are excluded.


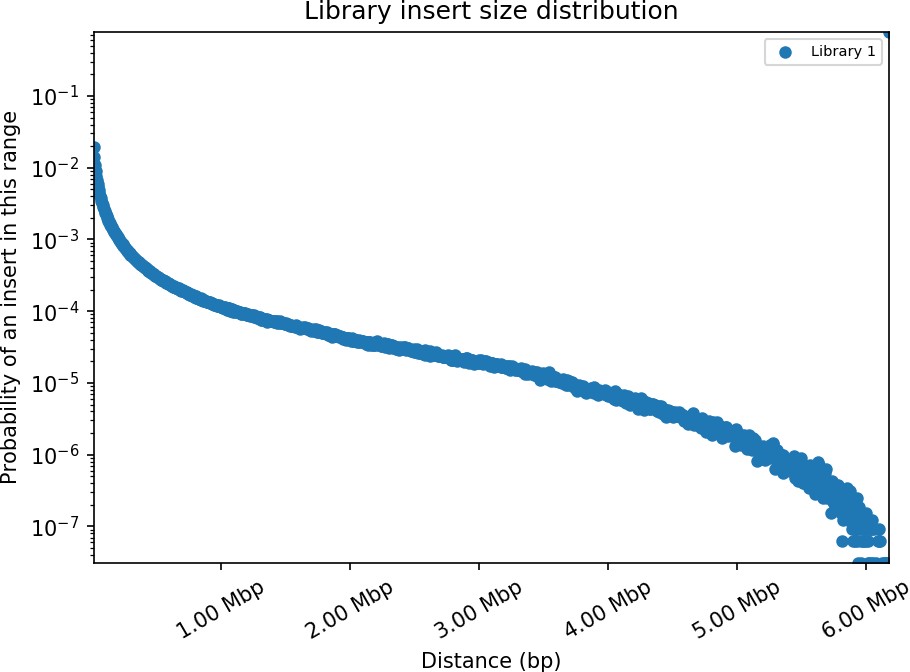


This figure shows the distribution of insert sizes in the Dovetail library. The distance between the forward and reverse reads is given on the X-axis in basepairs, and the probability of observing a read pair with a given insert size is shown on the Y- axis.

Comparative Assembly Statistics

|  | Input Assembly | Dovetail HiRise Assembly |
| --- | --- | --- |
| Longest Scaffold | 6,175,013 bp | 33,785,150 bp |
| Number of scaffolds | 12,788 | 8,017 |
| Number of scaffolds > 1kb | 12,786 | 8,015 |
| Contig N50 | 22.80 kb | 22.82 kb |
| Number of gaps | 35,383 | 40,123 |
| Percent of genome in gaps | 0.85% | 0.96% |
| * Note: Every join made by HiRise creates a gap. | | |
|  | Other Statistics |  |
| Number of breaks made to input assembly by HiRise | | 0 |
| Number of joins made by HiRise | | 4,771 |
| Number of gaps closed after HiRise | | 31 |
| Library 1 stats | | 226M read pairs; 2x150 bp |

BUSCO Stats

|  | Single copy | Duplicated | Fragmented | Missing | Total |
| --- | --- | --- | --- | --- | --- |
| Input Assembly | 236 | 1 | 27 | 39 | 303 |
| Dovetail HiRise Assembly | 236 | 1 | 26 | 40 | 303 |

Number of BUSCO (Benchmarking Universal Single-Copy Ortholog) genes found in the assembly before and after HiRise using the eukaryota odb9 dataset. Genes are split into four categories: complete and single-copy, complete and duplicated, fragmented, and missing.

**Glossary**

**Sequence Coverage** - For a given position in the genome, the sequence coverage is the number of times this basepair is directly observed in the sequencing data. Typically given as an average over the whole genome, or estimated by the total length of reads divided by the genome size.

**Physical Coverage** - For a given position in the genome, the physical coverage is the number of read pairs that span this position. Typically given as an average over the whole genome, or estimated by the area under the insert distribution divided by the genome size.

**Contig -** A contiguous genomic sequence without any gaps in an assembly.

**Scaffold** - A genomic sequence consisting of contigs that have been ordered and oriented relative to each other.

Contigs within scaffolds are separated by gaps (indicated by stretches of Ns).

**N50** - The scaffold length such that the sum of the lengths of all scaffolds of this size or larger is equal to 50% of the total assembly length.

**N90** - The scaffold length such that the sum of the lengths of all scaffolds of this size or larger is equal to 90% of the total assembly length.

**L50** - The smallest number of scaffolds that make up 50% of the total assembly length.

**L90** - The smallest number of scaffolds that make up 90% of the total assembly length.

**Supplementary Material G:** Circularized mitochondrial genome of *A. g. grandis* generated by NOVOplasty using mitochondrial reads pulled from a preliminary whole genome shotgun sequencing attempt of a single individual full body sample. Protein-coding genes are indicated in red, transfer RNAs (tRNAs) in blue, and ribosomal RNAs (rRNAs) in green. Note the tRNA Isoleucine (trnI) rearrangement into the otherwise non-coding control region on the right side of the figure.

**Supplementary Material H:** Analysis of molecular variance (AMOVA) and Monte Carlo permutation test results. D. F. is the degrees of freedom. % variation is the percentage of the variation in the data explained by the corresponding hierarchical level.

|  | **AMOVA results** | | | **Components of covariance** | | **MC perm.** |
| --- | --- | --- | --- | --- | --- | --- |
|  | **D. F.** | **Sum sq.** | **Mean sq.** | **Sigma** | **% variation** | **P-value** |
| **Between sequencing year** | 1 | 1168.753 | 1168.75286 | 1.699784 | 2.619333 | 0.2164 |
| **Between collections within seq. year** | 18 | 15648.051 | 869.33614 | 29.111038 | 44.859529 | 0.0001 |
| **Between samples within collections** | 272 | 10148.245 | 37.30972 | 3.226779 | 4.972402 | 0.0001 |
| **Within samples** | 292 | 9010 | 30.85616 | 30.856164 | 47.548735 | 0.0001 |
| **Total** | 583 | 35975.048 | 61.70677 | 64.893765 | 100 |  |


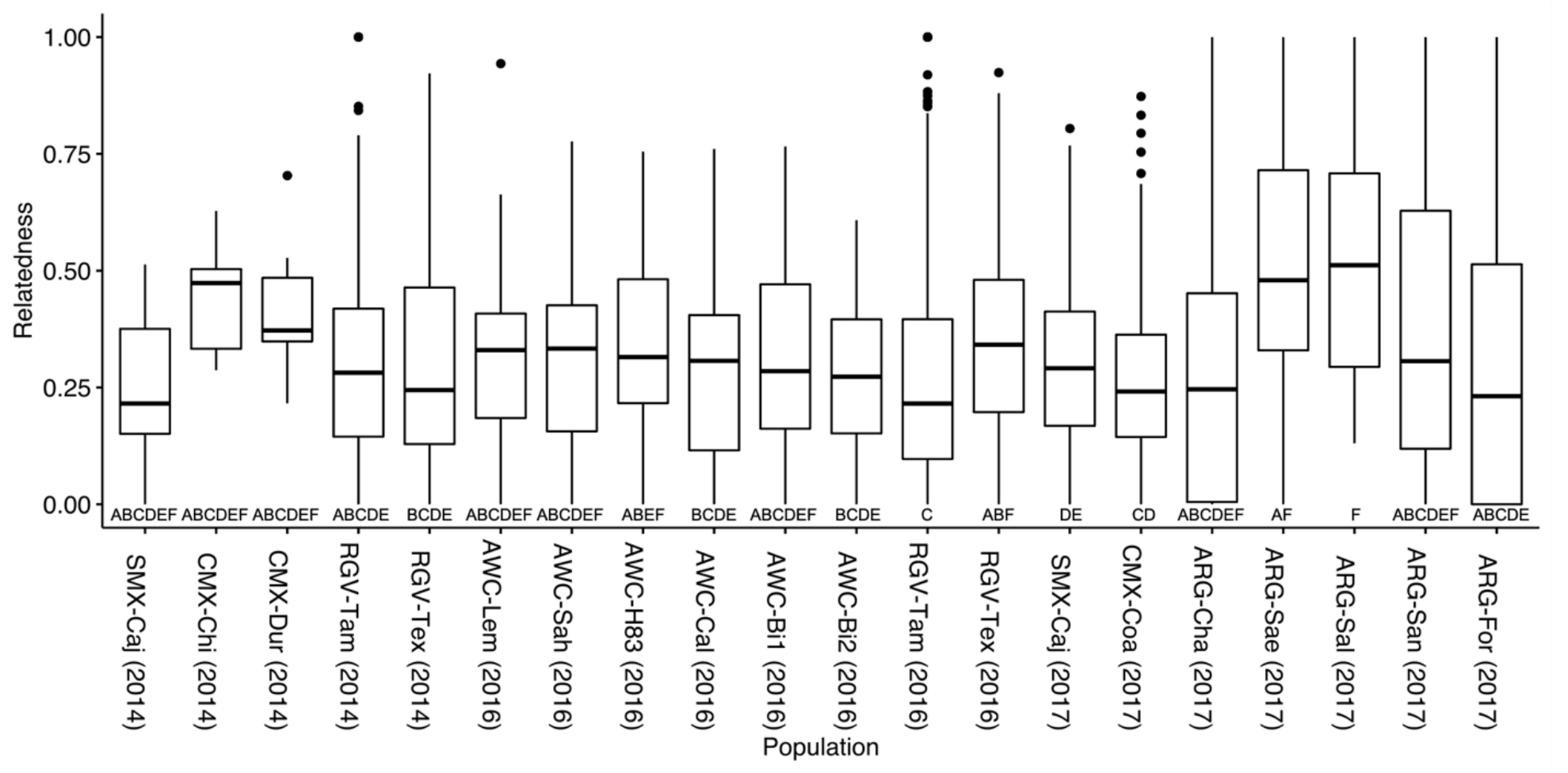


**Supplementary Material I:** Box and whisker plot of relatedness within populations. Kruskal-Wallis p < 0.0001. Wilcoxon rank test groups are shown beneath the lower whisker.

**Supplementary Material J:** Semi-matrices of pairwise comparisons of linear geographic distance (km, above the diagonal) and genetic distance (F_ST_, below the diagonal). Superscripts on the values in the F_ST_ semi-matrix indicate the results of the exact conditional contingency table tests of population differentiation (NS indicates P > 0.05 (not significant); all other pairwise comparisons are found to be statistically significant).

| **SMX-Caj (2014)** | - | 444.78 | 667.06 | 1196.52 | 1250.73 | 549.90 | 511.43 | 507.65 | 483.52 | 452.21 | 447.37 | 1197.53 | 1213.87 | 12.61 | 716.88 | 7984.80 | 8027.09 | 7675.75 | 8048.82 | 7928.97 |
| --- | --- | --- | --- | --- | --- | --- | --- | --- | --- | --- | --- | --- | --- | --- | --- | --- | --- | --- | --- | --- |
| **CMX-Chi (2014)** | 0.5207 | - | 326.23 | 793.80 | 840.30 | 661.94 | 644.38 | 632.56 | 637.83 | 550.67 | 535.61 | 794.68 | 803.34 | 449.76 | 380.00 | 7765.56 | 7801.03 | 7459.02 | 7845.84 | 7688.04 |
| **CMX-Dur (2014)** | 0.4478 | 0.2652 | - | 535.54 | 594.05 | 987.19 | 968.10 | 956.63 | 959.54 | 874.82 | 860.07 | 536.62 | 558.10 | 666.55 | 54.58 | 7439.32 | 7474.84 | 7132.80 | 7519.92 | 7362.31 |
| **RGV-Tam (2014)** | 0.3617 | 0.3299 | 0.2729 | - | 66.12 | 1423.15 | 1414.20 | 1401.22 | 1414.09 | 1321.41 | 1305.48 | 1.18 | 44.48 | 1197.10 | 492.82 | 7082.45 | 7110.51 | 6780.09 | 7180.21 | 6982.71 |
| **RGV-Tex (2014)** | 0.3493 | 0.3213 | 0.2665 | 0.0049 ^NS^ | - | 1459.16 | 1451.78 | 1438.64 | 1452.96 | 1359.62 | 1343.63 | 64.94 | 37.05 | 1251.70 | 553.20 | 7069.33 | 7096.15 | 6767.80 | 7169.83 | 6965.81 |
| **AWC-Lem (2016)** | 0.2544 | 0.6356 | 0.5932 | 0.5191 | 0.5056 | - | 41.59 | 42.30 | 75.39 | 115.00 | 128.33 | 1423.86 | 1423.60 | 562.07 | 1041.38 | 8424.51 | 8460.99 | 8117.46 | 8501.69 | 8349.47 |
| **AWC-Sah (2016)** | 0.2627 | 0.6469 | 0.6027 | 0.5341 | 0.5203 | 0.0002 ^NS^ | - | 13.66 | 34.13 | 93.89 | 109.39 | 1414.94 | 1415.94 | 523.51 | 1022.49 | 8402.51 | 8439.61 | 8095.21 | 8478.29 | 8329.46 |
| **AWC-H83 (2016)** | 0.2427 | 0.6269 | 0.583 | 0.5224 | 0.5103 | 0.0151 ^NS^ | 0.0032 ^NS^ | - | 40.33 | 81.89 | 97.09 | 1401.95 | 1402.83 | 519.81 | 1010.98 | 8391.80 | 8428.75 | 8084.55 | 8467.92 | 8318.29 |
| **AWC-Cal (2016)** | 0.2327 | 0.6129 | 0.5714 | 0.5028 | 0.4897 | -0.0106 ^NS^ | 0.004 ^NS^ | 0.0053 ^NS^ | - | 94.56 | 110.56 | 1414.84 | 1416.91 | 495.44 | 1014.05 | 8390.23 | 8427.88 | 8082.70 | 8464.72 | 8318.96 |
| **AWC-Bi1 (2016)** | 0.2603 | 0.6335 | 0.5927 | 0.5287 | 0.5155 | 0.0207 ^NS^ | 0.019 ^NS^ | 0.0126 ^NS^ | 0.0121 ^NS^ | - | 16.04 | 1322.15 | 1323.66 | 464.70 | 929.15 | 8310.63 | 8347.40 | 8003.46 | 8387.25 | 8236.65 |
| **AWC-Bi2 (2016)** | 0.2686 | 0.641 | 0.5988 | 0.5308 | 0.5174 | 0.0328 ^NS^ | 0.0375 ^NS^ | 0.0145 ^NS^ | 0.0304 ^NS^ | 0.0069 ^NS^ | - | 1306.23 | 1307.68 | 459.91 | 914.37 | 8296.46 | 8333.10 | 7989.35 | 8373.40 | 8222.09 |
| **RGV-Tam (2016)** | 0.3406 | 0.3275 | 0.2814 | 0.0351 | 0.0305 | 0.4835 | 0.4998 | 0.4922 | 0.4712 | 0.4964 | 0.4967 | - | 43.55 | 1198.12 | 493.92 | 7082.12 | 7110.16 | 6779.77 | 7179.92 | 6982.31 |
| **RGV-Tex (2016)** | 0.4863 | 0.4897 | 0.4672 | 0.2697 | 0.2672 | 0.5908 | 0.6018 | 0.5949 | 0.577 | 0.595 | 0.5986 | 0.2273 | - | 1214.88 | 517.91 | 7098.18 | 7125.44 | 6796.34 | 7197.68 | 6995.97 |
| **SMX-Caj (2017)** | 0.0752 | 0.5085 | 0.4662 | 0.4173 | 0.407 | 0.1706 | 0.1814 | 0.173 | 0.1577 | 0.1862 | 0.1984 | 0.3964 | 0.5008 | - | 715.95 | 7977.15 | 8019.61 | 7668.05 | 8040.76 | 7921.90 |
| **CMX-Coa (2017)** | 0.516 | 0.305 | 0.0931 | 0.375 | 0.3712 | 0.6111 | 0.6223 | 0.6099 | 0.5989 | 0.6168 | 0.6196 | 0.3544 | 0.502 | 0.5069 | - | 7385.72 | 7421.05 | 7079.28 | 7466.79 | 7308.19 |
| **ARG-Cha (2017)** | 0.6293 | 0.7432 | 0.6727 | 0.3778 | 0.363 | 0.6597 | 0.6742 | 0.6608 | 0.641 | 0.6595 | 0.6635 | 0.3244 | 0.4733 | 0.5213 | 0.592 | - | 113.59 | 310.36 | 260.71 | 348.72 |
| **ARG-Sae (2017)** | 0.6036 | 0.7392 | 0.6501 | 0.3516 | 0.3398 | 0.6441 | 0.6585 | 0.6432 | 0.6234 | 0.6428 | 0.6478 | 0.3039 | 0.4542 | 0.5072 | 0.5739 | 0.0058 ^NS^ | - | 375.71 | 359.25 | 258.67 |
| **ARG-Sal (2017)** | 0.6363 | 0.7773 | 0.6793 | 0.3921 | 0.382 | 0.666 | 0.6794 | 0.6628 | 0.6454 | 0.6632 | 0.6675 | 0.3422 | 0.4797 | 0.5246 | 0.5839 | 0.0763 ^NS^ | 0.0589 ^NS^ | - | 434.15 | 448.18 |
| **ARG-San (2017)** | 0.609 | 0.7495 | 0.6554 | 0.3626 | 0.3495 | 0.6482 | 0.6625 | 0.6473 | 0.628 | 0.6468 | 0.6516 | 0.315 | 0.4638 | 0.5086 | 0.5756 | 0.0041 ^NS^ | -0.0003 ^NS^ | 0.0302 ^NS^ | - | 609.05 |
| **ARG-For (2017)** | 0.6279 | 0.7436 | 0.6723 | 0.3883 | 0.3715 | 0.6565 | 0.6715 | 0.6583 | 0.6387 | 0.6569 | 0.6606 | 0.3307 | 0.4784 | 0.5161 | 0.5884 | 0.0288 ^NS^ | 0.0274 ^NS^ | 0.0992 | 0.0226 ^NS^ | - |
|  | **SMX-Caj (2014)** | **CMX-Chi (2014)** | **CMX-Dur (2014)** | **RGV-Tam (2014)** | **RGV-Tex (2014)** | **AWC-Lem (2016)** | **AWC-Sah (2016)** | **AWC-H83 (2016)** | **AWC-Cal (2016)** | **AWC-Bi1 (2016)** | **AWC-Bi2 (2016)** | **RGV-Tam (2016)** | **RGV-Tex (2016)** | **SMX-Caj (2017)** | **CMX-Coa (2017)** | **ARG-Cha (2017)** | **ARG-Sae (2017)** | **ARG-Sal (2017)** | **ARG-San (2017)** | **ARG-For (2017)** |

**Supplementary Material K:** Summarized pairwise F_ST_ table showing the ranges of F_ST_ values for each of five major geographic sampling regions across multiple years.

| **SMX (2014)** | - |  |  |  |  |  |  |  |
| --- | --- | --- | --- | --- | --- | --- | --- | --- |
| **CMX (2014)** | 0.45 - 0.52 | 0.27 |  |  |  |  |  |  |
| **RGV (2014)** | 0.35 - 0.36 | 0.27 - 0.33 | 0.00 |  |  |  |  |  |
| **AWC (2016)** | 0.23 - 0.27 | 0.57 - 0.65 | 0.49 - 0.53 | -0.01 - 0.04 |  |  |  |  |
| **RGV (2016*)*** | 0.34 - 0.49 | 0.28 - 0.49 | 0.03 - 0.27 | 0.47 - 0.60 | 0.23 |  |  |  |
| **SMX (2017)** | 0.08 | 0.47 - 0.51 | 0.41 - 0.42 | 0.16 - 0.20 | 0.40 - 0.50 | - |  |  |
| **CMX (2017)** | 0.52 | 0.09 - 0.31 | 0.37 - 0.38 | 0.60 - 0.62 | 0.35 - 0.50 | 0.51 | - |  |
| **ARG (2017)** | 0.60 - 0.64 | 0.65 - 0.78 | 0.34 - 0.39 | 0.62 - 0.68 | 0.30 - 0.48 | 0.51 - 0.52 | 0.57 - 0.59 | 0.00 - 0.10 |
|  | **SMX (2014)** | **CMX (2014)** | **RGV (2014)** | **AWC (2016)** | **RGV (2016*)*** | **SMX (2017)** | **CMX (2017)** | **ARG (2017)** |


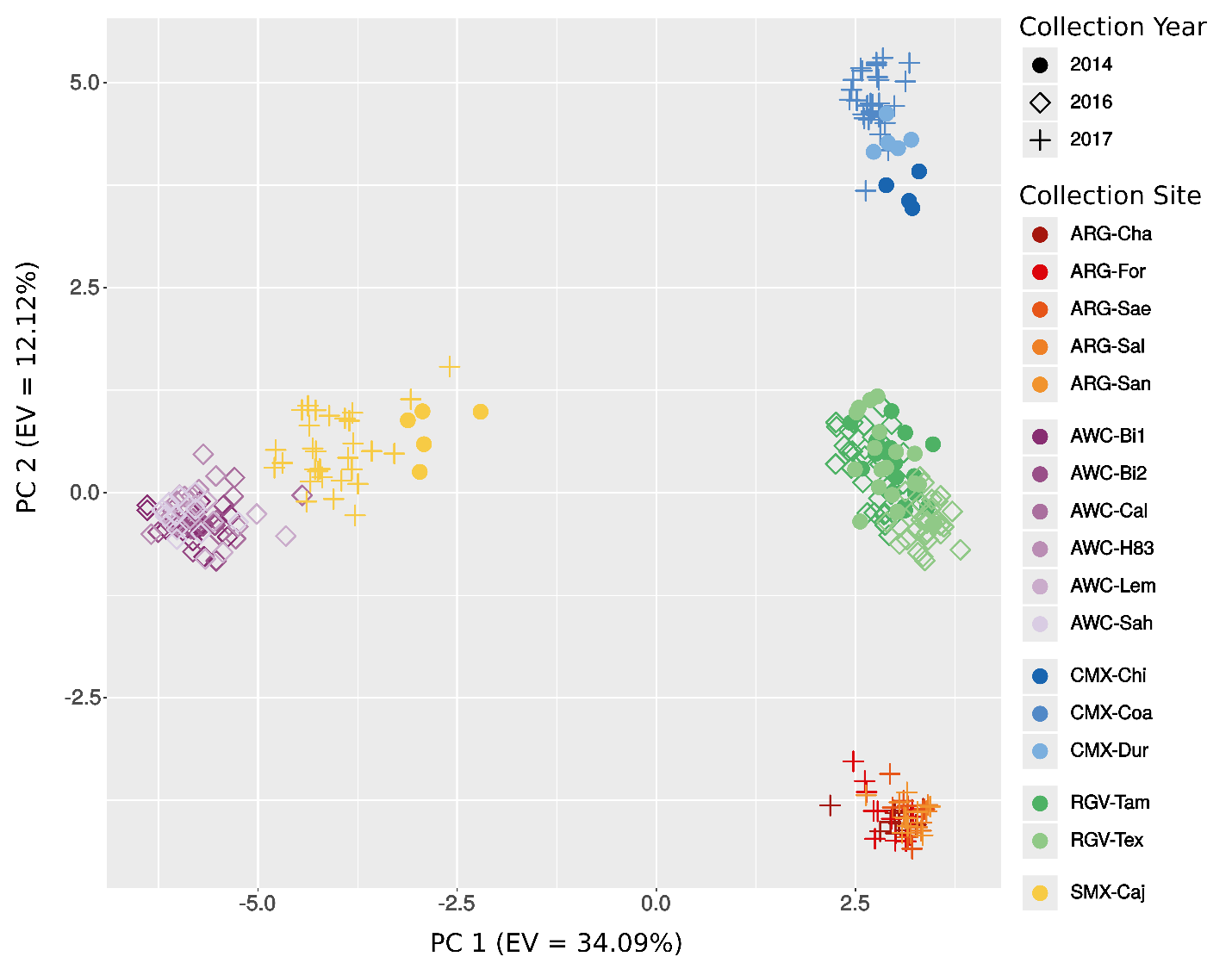


**Supplementary Material L:** Results of principal components analysis as performed using R/adegenet. Principal component 2 is plotted as a function of principal component 1. EV are eigenvalues. Geographic locality is denoted by color and collections from the same site but from across multiple years are differentiated by different symbols.
